# Exploration of normal placental blood flow characteristics in middle and late pregnancy based on microvascular flow imaging

**DOI:** 10.12669/pjms.41.7.12135

**Published:** 2025-07

**Authors:** Chuanfen Gao, Chaoxue Zhang, Ying Zhang, Yi Zhou, Xiufang Shuai, Lin Gao

**Affiliations:** 1 Chuanfen Gao, Department of Ultrasound, The First Affiliated Hospital of Anhui Medical University, Hefei, Anhui Province 230022, P.R. China; 2 Chaoxue Zhang, Department of Ultrasound, The First Affiliated Hospital of Anhui Medical University, Hefei, Anhui Province 230022, P.R. China; 3 Ying Zhang, Department of Obstetrics, The First Affiliated Hospital of Anhui Medical University, Hefei, Anhui Province 230022, P.R. China; 4 Yi Zhou, Department of Ultrasound, The First Affiliated Hospital of Anhui Medical University, Hefei, Anhui Province 230022, P.R. China; 5 Xiufang Shuai, Department of Ultrasound, The First Affiliated Hospital of Anhui Medical University, Hefei, Anhui Province 230022, P.R. China; 6 Lin Gao, The First of Clinical College of Anhui Medical University, Hefei 230022, Anhui Province, China

**Keywords:** Micro blood flow, MV-Flow, Middle and late pregnancy, Placenta, Singleton pregnancy

## Abstract

**Objective:**

To explore the micro blood flow characteristics of normal singleton pregnancy placenta in middle and late pregnancy based on microvascular flow (MV-Flow) imaging.

**Methods:**

We conducted a retrospective cohort study. Clinical records and placental ultrasound images of 485 patients in mid to late pregnancy with normal fetal development who underwent MV-Flow microvascular imaging in The First Affiliated Hospital of Anhui Medical University from January 2023 to December 2023 were retrospectively analyzed. The gestational age of patients ranged from 22 to 38 weeks, and the biological measurements of the fetus during pregnancy were within the normal range. The patients were divided into six groups according to gestational age: 22-24W, 25-27W, 28-30W, 31-33W, 34-36W, and 37-39W.

**Results:**

During the middle and late stages of normal pregnancy, the placental microvasculature displayed grade three or higher blood flow branches. As the gestational age increased, the peak systolic velocity (PSV) of the fetal umbilical artery (UA) and middle cerebral artery (MCA) gradually increased. The UA pulsatility index (PI), MCA-PI, and ductus venosus (DV) - PI gradually decreased with the gestational age (all *P*<0.05). There were statistically significant differences in the PSV and PI of various branches of the chorionic vascular tree in different gestational age groups (all *P*<0.05). However, there was no statistically significant difference in vascular index from MV-Flow (VI^MV^) (*P*>0.05), with no significant changes at different gestational weeks.

**Conclusions:**

Placental microvascular imaging based on MV-Flow can demonstrate a gradual increase in the microvascular flow velocity inside the placenta and a gradual decrease in the blood flow resistance with gestational age.

## INTRODUCTION

Abnormal placental blood perfusion can cause intrauterine fetal growth restriction.^1^,^2^ Currently, fetal circulatory hemodynamics is mainly evaluated through pulse Doppler ultrasonography (US) of the umbilical and middle cerebral arteries.^3^,^4^ However, displaying the microvascular structural characteristics of the placenta using the conventional color Doppler and power Doppler US is challenging.^4^ The traditional Doppler techniques are limited in exploring the microvascular structure of the placenta and cannot fully display the normal placental microvasculature.^3^–^5^ In addition, there are limitations to directly quantifying placental or placental bed blood vessels using a combination of three-dimensional (3D) imaging and Doppler US, as these techniques cannot effectively distinguish between fetal circulation within the placenta and maternal-fetal perfusion blood flow.^4^,^5^

Although contrast-enhanced Ultrasound can display placental blood flow perfusion, its safety during pregnancy is not guaranteed.^5^,^6^ Partial placental vascular imaging techniques have high-resolution capabilities, but their application is limited.^6^ The ultra-fast Doppler technology can form a highly sensitive map of placental blood vessels without the need to inject contrast agents.^7^ It can distinguish between maternal and fetal blood based on the pulsatile behavior of maternal and fetal blood. At present, this method is applied in the rabbit placenta test.^7^ While microscopic computer tomography (CT), optical microscopy, and electron microscopy provide three-dimensional imaging of placental structures with the true spatial geometry of placental blood vessels, their use is limited to *in vitro* studies.^8^ The changes in placental blood flow before delivery still need further exploration.

Microvascular flow (MV-Flow) is an advanced power Doppler technique.^9^ While the traditional color or power Doppler cannot detect blood flow with velocities below a constant (<200 Hz), MV-Flow provides a clear view of low-speed blood flow related to surrounding tissues, with high sensitivity and resolution and can detect microvessels entering tissues and organs.^9^,^10^ This imaging technology has been applied to investigate various aspects of fetal development, such as the fetal brain, ovarian tumors, renal cystic lesions, and skin.^9^–^11^ However, limited data are available of this technology on normal placental blood flow characteristics in middle and late pregnancy. Therefore, this study explored the microvascular perfusion imaging of the placenta during normal mid to late pregnancy using MV-Flow imaging and analyzes its blood flow characteristics.

## METHODS

Clinical records of 485 women with singleton pregnancies and normal fetal development between 22 and 38 weeks of gestation who underwent MV-Flow examination in The First Affiliated Hospital of Anhui Medical between January 2023 to December 2023 were retrospectively analyzed. Baseline characteristics of all women, including age, placental position, placental thickness, fetal weight percentile, and pregnancy complications, were collected. The cohort was divided into six groups according to gestational age: 22-24W, 25-27W, 28-30W, 31-33W, 34-36W, and 37-39W. Determination of gestational age was based on the regular last menstrual period or in vitro fertilization transfer date. For women with irregular menstruation, the gestational age was calculated by measuring the head hip diameter during a nuchal translucency (NT) examination at 11-13 weeks and six days.

### Ethical approval:

All procedures performed in the study involving human participants followed the ethical standards of the institutional and/or national research committee(s) and the Helsinki Declaration. The Ethical Committee of the First Affiliated Hospital of Anhui Medical has approved the protocol (No. Quick-PJ 2023-14-43, Date: December 14^th^, 2023), and the need for informed consent was waived due to the observational and retrospective nature of the study. All data were stored securely, and confidentiality was maintained throughout the study.

### Inclusion criteria:

- Pregnancy from 22 weeks to 38 weeks.
- Single natural pregnancy with normal placenta morphology and with normal fetal growth and development.
- Healthy pregnant women without pregnancy complications.

### Exclusion criteria:

- Chromosomal or genetic fetal abnormalities.
- Fetal structural abnormalities indicated by prenatal US or magnetic resonance imaging examination;
- Membranous placenta, extensive calcification or multiple infarctions in the placenta, and concomitant placental choriocarcinoma.
- Excessive amniotic fluid, thick abdominal fat in pregnant women, or uterine adenomyosis and fibroids in pregnant women that can interfere with the clear display of placental microcirculation.

### MV-Flow ultrasound imaging:

Samsung HERA W10 (Samsung, South Korea) color Doppler US diagnostic instrument was used. The abdominal convex array probe was set at a frequency of 2-9MHz. All imaging and Doppler US examinations were performed under default settings, adopting the elliptical shape of the region of interest (ROI) size provided by the instrument. MV-Flow was activated, and the image was optimized to obtain a clear placental villous vascular tree image. LumiFlow was activated to make blood flow display more three-dimensional. MV-Flow parameter settings were standardized using the HERA W10 system.

The settings were as follows: image quality, normal; Sensitivity setting 26; Organizational inhibition setting three; Set the color gain to 50; Filter setting three; Smooth setting one; LumiFlow setting two; Set alpha blending to 75%; Set dynamic range to 20; Pseudo color map setting two. The size of the sampling box was carefully defined, including the thickness of the placental parenchyma. Care was taken not to exceed the placenta fetal plane and maternal plane as much as possible, and to prevent interference from umbilical cord blood flow near the fetal surface of the placenta and maternal uterine wall blood flow. A fixed size ROI was maintained and the placental vascular index (VI^MV^) was obtained based on MV-Flow. The technologist searched for the clearest location on the placental villous vascular tree and took a sample to save the image.

The ROI area (in cm^2^) and VI^MV^ value percentage were recorded. The images were taken while the woman was breathing quietly, and the fetus was moving as little as possible to maintain image stability. The placental image was enlarged and the sampling box size was adjusted. The chorionic vascular tree and chorionic plate vessels were displayed as much as possible, and chorionic plate vessels (CPV), primary villi vessels (PVV), secondary villi vessels (SVV), and tertiary villi vessels (TVV) in the placenta were identified. The PSV and PI of CPV, PVV, SVV, and TVV were measured separately. All cases were examined by ultrasound technologists engaged in prenatal screening for over five years.

### Observation indicators:

- Blood flow characteristics of uterine artery, middle cerebral artery and umbilical artery at different gestational weeks.
- Blood flow characteristics of different levels of chorionic vascular branches at different gestational weeks.
- Trend of flow velocity changes in various levels of villial branches in different pregnancy groups.
- Trend of PI changes in various levels of villous branch blood vessels in different gestational age groups.

### Statistical methods:

All data analyses were conducted according to a predefined statistical analysis plan. The data was analyzed using SPSS 22.0 software (IBM, Armonk, New York, USA). The measurement data were expressed as mean ± standard deviation (SD). The repeated measures analysis of variance (ANOVA) was used to examine data from different pregnancy stages. *P*<0.05 was considered statistically significant.

## RESULTS

**Fig.1:**
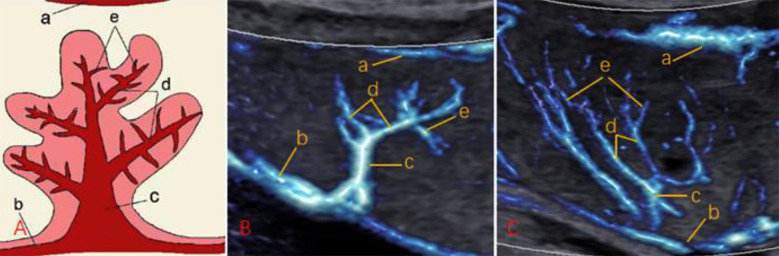
Placental villus blood flow patterns and ultrasound MV-Flow images at 22 weeks. (A) The villi’s contour and vascular pattern, a normal placenta functional unit. The background color in the picture represents the placental parenchyma, the blue tree-like structure represents a villous unit, and the red tree-like structure represents the villous vascular tree. (B) Images of placental villi evaluated by MV-Flow imaging at 22 weeks of pregnancy. Corresponding to the pattern diagram in (A), the low echo blue dendritic echo inside the placenta is the placental villous vascular tree. (C) MV-Flow imaging evaluation of the placental villi at 28 weeks of gestation. Corresponding to the pattern diagram in (A), the number of fuzzy branches increases and becomes longer. a represents placental basal plate vessels; b represents CPV; c represents PVV; d represents SVV; e represents TVV.

Baseline clinical characteristics of 485 pregnant women are shown in Table-I. As shown in Fig.1, MV-Flow could clearly display the branches of the chorionic vascular tree and the umbilical artery branch entering the submucosa and becoming a CPV. CPV entered the placenta to form PVV; PVV branched out to form SVV; SVV continued to branch and form TVV. MV-Flow could distinguish placental basal plate vessels, CPV, PVV, SVV, and TVV. Fig.1

**Table-I:** Basic clinical characteristics.

	n(%)/mean±SD
Age (years), mean±SD	30.49±4.39
***Placental position, n(%)***	
Side wall	16 (3.3)
Palace floor	14 (2.9)
Rear wall	169 (35.1)
Front wall	283 (58.7)
Placental thickness (mm), mean±SD	35.95±11.16
Estimate weight percentile, mean±SD	43.67±23.28
***Complication, n(%)***	
Nothing	291 (60.0)
Anemia	93 (19.2)
Diabetes	65 (13.4)
Hypothyroidism	53 (11.0)
Hypertension	16 (3.3)
Coagulation disorders	1 (0.2)
Systemic lupus erythematosus	1 (0.2)

**Fig.2:**
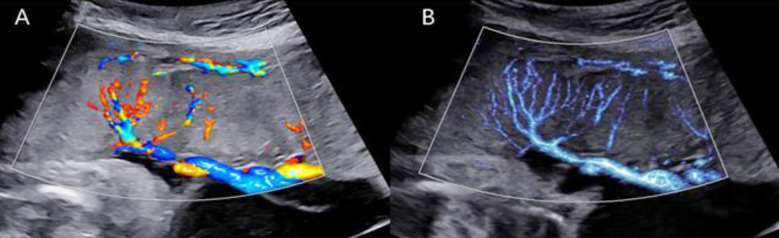
Different flow imaging patterns of the normal placenta (30 weeks). (A) A color Doppler flow chart with a low range (scale 9.6cm/s) displaying CPV, PVV, SVV, some TVV, and placental basal plate vessels, with color flow showing overflow; (B) The microvasculature of the same placental section, with clear visualization of the third or higher branches of the placental villous vascular tree.

**Fig.3:**
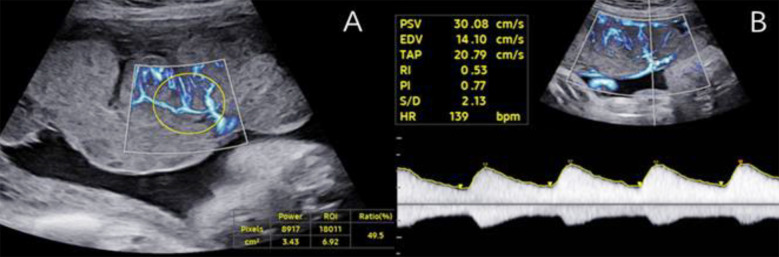
Measurement of VI^MV^ and PVV in normal placental villous vascular tree. (A) VI^MV^ measurement; (B) PVV blood flow spectrum.

As shown in Fig.2, MV-Flow clearly displayed blood flow, with no overflow of blood flow in the branch vessels. The display, in general, and the display of low-speed blood flow in the distal branch, in particular, were comprehensive. Fig.3 illustrates the MV-flow measurement of VI^MV^ and branch vessels in a normal placental villous vascular tree using spectral Doppler. The sampling box was enlarged to fully display and measure the peak systolic velocity (PSV) and pulsatility index (PI) of CPV, PVV, SVV, and TVV.

The cohort was divided into six groups according to gestational age, and the PSV and PI of umbilical artery (UA), middle cerebral artery (MCA), uterine artery (Ura) PI, and ductus venosus (DV) in different gestational age groups were compared. As shown in Table-II, the differences in UA-PSV, UA-PI, MCA-PSV, MCA-PI, LUrA PI, and DV-PI were statistically significant (all *P*<0.05). As the gestational age increased, UA-PSV and MCA-PSV gradually increased, while UA-PI, MCA-PI, LUrA PI, and DV PI gradually decreased (all *P*<0.05) Table-II.

**Table-II:** Blood flow characteristics of uterine artery, middle cerebral artery and umbilical artery at different gestational weeks.

	22-24W (n=87)	25-27W (n=53)	28-30W (n=129)	31-33W (n=108)	34-36W (n=68)	37-39W (n=37)	F	P
UA-PSV	38.57±7.61	41.79±7.69	42.93±7.59	46.11±8.95	49.79±9.71	51.38±12.33	18.24	<0.001
UA-PI	1.07±0.21	0.99±0.23	0.93±0.21	0.89±0.23	0.87±0.19	0.71±0.16	18.81	<0.001
MCA-PSV	29.44±5.10	35.34±6.13	41.63±8.34	49.31±9.98	57.24±9.64	62.76±13.94	158.06	<0.001
MCA-PI	1.76±0.37	1.91±0.48	2.03±0.46	1.95±0.44	1.65±0.40	1.47±0.47	15.15	<0.001
LUrA-PI	0.83±0.27	0.71±0.20	0.76±0.31	0.71±0.23	0.73±0.26	0.66±0.16	3.59	0.003
RUrA-PI	0.76±0.27	0.76±0.30	0.72±0.27	0.70±0.33	0.68±0.21	0.61±0.17	2.14	0.060
DV-PI	0.59±0.24	0.51±0.20	0.52±0.22	0.50±0.23	0.43±0.24	0.49±0.29	3.70	0.003

UA-PSV: peak systolic velocity of umbilical artery, UA-PI: pulsatility index of umbilical artery, MCA-PSV: peak systolic velocity of middle cerebral artery, MCA-PI: pulsatility index of middle cerebral artery, LUrA-PI: ulsatility index of left uterine artery, RUrA-PI: pulsatility index of right uterine artery, DV-PI: pulsatility index of ductus venosus.

Micro blood flow indicators such as CPV-PSV, CPV-PI, PVV-PSV, PVV-PI, SVV-PSV, SVV-PI, TVV-PSV, TVV-PI, and VI^MV^ were then measured at different gestational weeks. The results showed statistically significant gestation-associated differences in PSV and PI among the branches of the chorionic vascular tree at all levels (all *P*<0.05). There was no statistically significant difference in VI^MV^ (*P*>0.05). The results are shown in Table-III.

**Table-III:** Blood flow characteristics of different levels of chorionic vascular branches at different gestational weeks.

	22-24W (n=87)	25-27W (n=53)	28-30W (n=129)	31-33W (n=108)	34-36W (n=68)	37-39W (n=37)	F	P
CPV-PSV	16.15±6.87	19.29±7.37	20.63±7.54	21.9±8.01	22±8.69	21.52±7.44	7.08	<0.001
CPV-PI	1.06±0.22	0.97±0.18	0.96±0.19	0.9±0.18	0.85±0.15	0.77±0.19	19.07	<0.001
PVV-PSV	13.67±4.55	19.59±11.85	18.23±6.03	18.96±5.97	19.96±5.99	17.2±5.93	16.14	<0.001
PVV-PI	0.96±0.18	0.85±0.14	0.85±0.16	0.83±0.18	0.76±0.22	0.67±0.14	17.60	<0.001
SVV-PSV	9.53±2.84	12.3±5.68	12.16±3.41	13.15±4.06	13.77±4.15	12.94±4.67	17.13	<0.001
SVV-PI	0.89±0.24	0.82±0.22	0.83±0.47	0.77±0.19	0.69±0.16	0.64±0.13	6.17	<0.001
TVV-PSV	6.07±1.93	7.3±2.74	7.57±2.17	8.34±2.83	8.78±3.36	8.59±2.89	11.62	<0.001
TVV-PI	0.76±0.2	0.69±0.17	0.72±0.23	0.67±0.18	0.61±0.14	0.53±0.13	10.66	<0.001
VI^MV^	59.49±13.92	58.75±12.54	61.19±11.91	59.57±14.09	61.32±11.67	59.8±12.97	0.50	0.776

CPV-PSV: peak systolic velocity of chorionic plate vessels, CPV-PI: pulsatility index of chorionic plate vessels, PVV-PSV: peak systolic velocity of primary villi vessels, PVV-PI: pulsatility index of primary villi vessels, SVV-PSV: peak systolic velocity of secondary villi vessels, SVV-PI: pulsatility index of secondary villi vessels, TVV-PSV: peak systolic velocity of tertiary villi vessels, TVV-PI: pulsatility index of tertiary villi vessels, VI^MV^ : vascular index based on MV-Flow.

**Fig.4:**
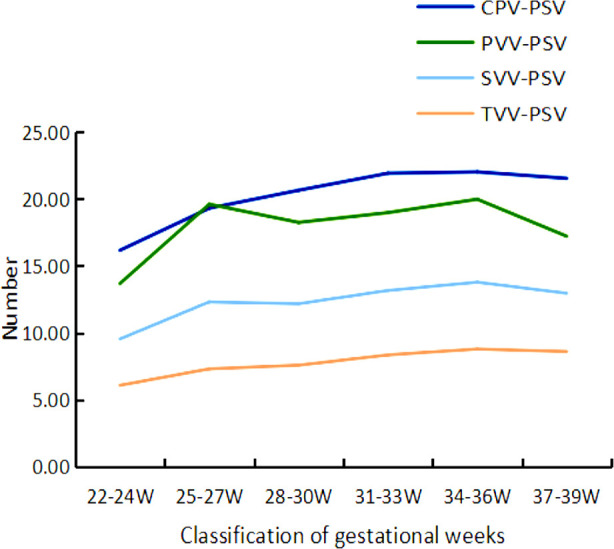
Trend of Flow Velocity Changes in Various Levels of Villial Branches in Different Pregnancy Groups.

**Fig.5:**
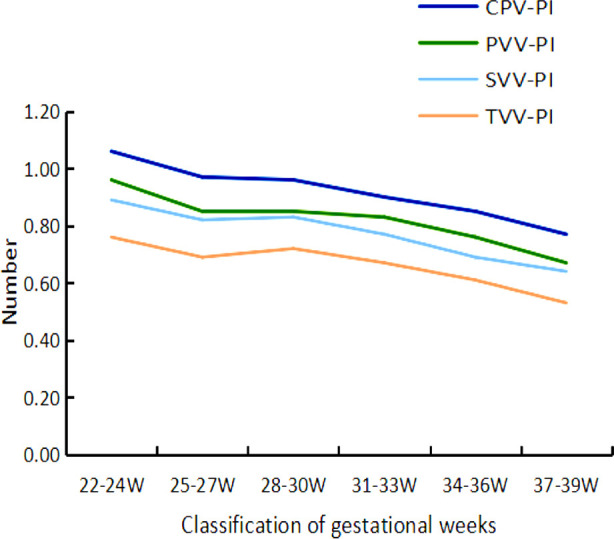
Trend of PI changes in various levels of villous branch blood vessels in different gestational age groups.

The flow velocity of various branches of blood vessels (CPV-PSV, PVV-PSV, SVV-PSV, TVV-PSV) showed an increasing trend with the increasing gestational age (Fig.4). The pulsatility index (CPV-PI, PVV-PI, SVV-PI, TVV-PI) of various branches of blood vessels decreased with increasing gestational age. Fig.5 The VI^MV^ showed no significant changes at different gestational weeks.

## DISCUSSION

This study assessed the blood flow characteristics of the normal placenta during late pregnancy based on MV-Flow imaging. The results showed that MV-Flow can display blood flow in the placental villous vascular tree and detect a gradual increase in the microvascular flow velocity inside the placenta and a gradual decrease in the blood flow resistance with gestational age.

Most current studies suggest that the final stage of villous vascular growth occurs between 24-26 weeks of pregnancy.^12^–^14^ This study selected microvasculature features that can stably display the tertiary branches of chorionic blood vessels starting from 22 weeks of pregnancy. The study found that the MV-Flow imaging can clearly display blood vessels of the normal placental chorionic villi, and their internal tertiary villous tree vessels in the middle and late stages of pregnancy. In addition to directly measuring its blood flow spectrum, VI^MV^ can also be used to quantify placental blood flow.

The results of this study showed that the maternal-fetal blood flow parameters of UA and MCA at different gestational weeks and the peak flow velocity gradually increased with the increase of gestational weeks. In contrast, vascular resistance parameters, such as UA, MCA, and DV, gradually decreased. These results align with fetal growth and development physiology, as fetal blood flow perfusion increases with gestational age.^15^,^16^ Similarly, this study showed that the branch blood vessels of the normally developing placental chorionic vascular tree gradually increased with the increase of gestational age. This is consistent with the developmental characteristics of the placenta, as the constantly increasing metabolic demands during fetal growth are matched with the increased placenta blood flow and growth.^15^–^17^

The results of this study also showed that the PSV and PI of various branches of the chorionic vascular tree in normal placenta, such as CPV, PVV, SVV, and TVV, varied at different gestational weeks. The PSV of various branches of blood vessels showed an increasing trend with gestational age, while the PI gradually decreased. Importantly, placental vascular index VI^MV^ showed no significant changes at different gestational weeks, indicating that after the stable growth and development of chorionic blood vessels, the branch blood flow displayed by ultrasound micro blood flow is relatively stable.^18^,^19^ There are reports^17^–^19^ that the vascular index of the normal placenta is higher than that of the placenta in pregnancies that are complicated by intrauterine fetal growth restriction (FGR). This difference may be due to insufficient development of peripheral blood vessels in the chorionic villi tree. This study aimed to demonstrate that the density of vascular distribution can be evaluated through the quantitative indicator VI^MV^ in the normally developing placenta, providing a theoretical reference for the mechanism of placental-derived FGR caused by placental vascular lesions.

In this study, the measurement of vascular index effectively distinguished the basal vessels and avoided the interference of maternal vascular lakes. Placental basal plate vessels are blood vessels that run along the maternal surface of the placenta, almost perpendicular to the branches of the chorionic villi tree, making them easy to identify.^20^ The blood flow morphology of the placental maternal vascular lake is mostly small irregular shapes, primarily located below or at the edge of the chorionic plate.^20^,^21^ They lack vascular branching characteristics, which do not conform to the characteristic of gradually thinning blood vessels at the end of the chorionic tree and can be, thus, distinguished.^21^,^22^ Yin et al^23^ have confirmed that MV-Flow can quantitatively assess placental blood perfusion in the first-trimester of pregnancy. However, limited data are available of this technology on normal placental blood flow characteristics in middle and late pregnancy. This study confirmed that MV Flow can clearly display the blood flow of the chorionic vascular tree in normal placenta during mid to late pregnancy. By early detection of abnormal placental blood flow, medical staff can take timely measures to reduce the risk of adverse pregnancy outcomes such as premature birth, fetal distress, and stillbirth.

### Limitations:

This is a retrospective study with a limited sample size. The microvascular structure of the placenta based on MV-Flow imaging only shows blood flow branches, lacking the display of blood flow direction. The junction between the terminal branches of the chorionic villi tree blood vessels inside the placenta and the maternal side blood vessel branches is easily confused. Recently, studies have used MV-Flow 3D ultrasound to display fine neural vessels in the fetal brain, which can enhance vascular imaging of the arteriovenous system.^19^,^24^ If three-dimensional microvascular imaging technology can be used to display the branches of placental microvessels, it will allow to explore its spatial distribution characteristics further. Further large-scale, prospective studies are needed to confirm the findings of this study and to establish a definitive conclusion.

## CONCLUSION

MV-Flow can clearly display the blood flow of the chorionic vascular tree in the normal placenta during mid to late pregnancy. As the gestational age increases, the flow velocity of various branches of blood vessels shows an increasing trend while the resistance gradually decreases. However, the vascular index does not show significant changes with increasing gestational age, which can provide an important reference for placental FGR characterized by the reduced placental blood supply.

### Authors’ contributions:

**CG:** Study design. Literature search and manuscript writing. Manuscript revision and validation and is responsible for the integrity of the study.

**CZ, YZ, Yi Zhou, XS and LG:** Data collection, data analysis and interpretation. Critical Review.

All authors have read and approved the final manuscript.
